# The mechanistic role of cardiac glycosides in DNA damage response and repair signaling

**DOI:** 10.1007/s00018-023-04910-9

**Published:** 2023-08-16

**Authors:** Diana Ainembabazi, Youwei Zhang, John J. Turchi

**Affiliations:** 1 Department of Medicine, School of Medicine, Joseph E Walther Hall, Indiana University, 980 W. Walnut St, C560, R3-C560, Indianapolis, IN 46202 USA; 2 Department of Pharmacology, School of Medicine, Case Western Reserve University, 10900 Euclid Avenue, Cleveland, OH 44106 USA

**Keywords:** Cardenolide, Bufadienolide, Cancer therapeutics, Cytotoxicity, Structure–activity relationships

## Abstract

Cardiac glycosides (CGs) are a class of bioactive organic compounds well-known for their application in treating heart disease despite a narrow therapeutic window. Considerable evidence has demonstrated the potential to repurpose CGs for cancer treatment. Chemical modification of these CGs has been utilized in attempts to increase their anti-cancer properties; however, this has met limited success as their mechanism of action is still speculative. Recent studies have identified the DNA damage response (DDR) pathway as a target of CGs. DDR serves to coordinate numerous cellular pathways to initiate cell cycle arrest, promote DNA repair, regulate replication fork firing and protection, or induce apoptosis to avoid the survival of cells with DNA damage or cells carrying mutations. Understanding the *modus operandi* of cardiac glycosides will provide critical information to better address improvements in potency, reduced toxicity, and the potential to overcome drug resistance. This review summarizes recent scientific findings of the molecular mechanisms of cardiac glycosides affecting the DDR signaling pathway in cancer therapeutics from 2010 to 2022. We focus on the structural and functional differences of CGs toward identifying the critical features for DDR targeting of these agents.

## Introduction

Cardiac glycosides (CGs) have traditionally been used to treat congestive heart failure and arrythmia; however, their pharmacological applications have been extended to other diseases including cancer, viral infection, inflammation, neurological, and autoimmune diseases [2–5]. The first therapeutic utility of CGs in cancer was reported in 1967 [6]. A decade later, Stenkvist et al*.* observed tumor reduction in breast cancer patients that had taken digoxin [7]. The study also noted that these patients had a lower risk of recurrence. Since then, the anti-cancer properties of CGs have generated considerable interest [8–12]. This includes a dramatic increase in the number of in vitro and in vivo studies evaluating the effects of cardiac glycosides on the inhibition of various types of cancer, demonstrating the ability to block cancer cell proliferation and subsequently cause cell death [13–17].

**Fig. 1:**
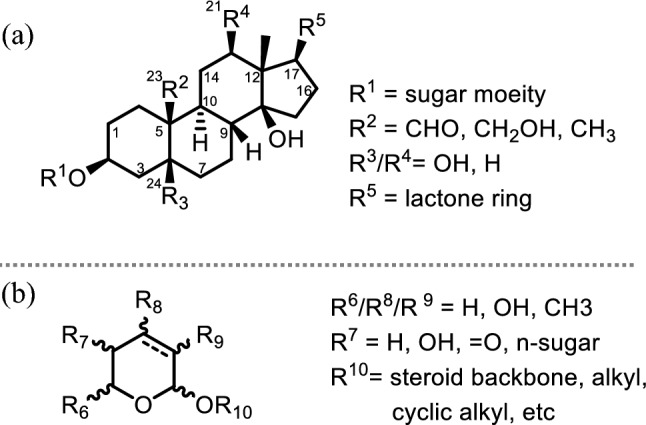
**a** Conventional structural framework of CGs and **b** Structural scaffold of glycosides considered

CGs belong to a class of diverse, naturally occurring bioactive compounds that are structurally comprised of a steroid core bound to a lactone ring at C17 and a sugar group at C2 (Fig. 1a). These structural motifs serve a critical purpose in the function of the compound by influencing the binding affinity, pharmacokinetics, and pharmacodynamics of the molecule [18–20]. The development of novel structural modifications and assessing their impact on function is essential in designing more potent anti-cancer CGs.

However, as important as investigating the structure–activity relationships (SARs) is in advancing CGs as potential anti-cancer drugs, understanding their molecular mechanism in biological systems is equally important. Unfortunately, the mechanism of cytotoxicity in cancer cells upon treatment with CGs has not yet been fully elucidated. Furthermore, while numerous studies have shown that CGs increase the sensitivity of cancerous cells when used in combination with current cancer therapies [14, 21, 22], the mechanisms of these drug–drug interactions are still not known. While these studies have provided treatment regimens to address drug resistance to first-line therapeutic drugs like cisplatin, how they achieve this synergy is just beginning to be elucidated. Therefore, there is a critical need to understand the anti-cancer mechanism of CGs both as single agents and in combination with existing therapies.

There have been several anti-cancer mechanisms of CG action reported in different cancer models. Prassas and Diamandis showed that the CGs inhibit Na^+^/K^+^ ATPase or activate the oncogenic Ras pathway which results in the generation of reactive oxygen species (ROS) and subsequent cell death [23]. Other modes of action explored include inhibiting the phosphatidylinositol 3-kinase (PI3K) pathway [24], activating endoplasmic reticulum stress, inhibiting hypoxia inducible factor 1 alpha (HIF-1α), and extracellular signal-regulated kinase 1/2 (ERK1/2) signaling pathway [25]. Growth signaling pathways have also been shown to be modulated by CG treatment with inhibition of the STAT-3 pathway [15, 26], increasing forkhead box O1 (FOXO1) expression [27], inhibiting NF-κB [16] OBJ, and activating MAPK-Nu77 signaling pathway [28]. Finally, effects on transcription, splicing, and translation have also been reported [29]. Multiple studies implicate the DNA damage response (DDR), and reduced expression of DNA repair proteins and kinases has been reported with GC treatment [30, 31], and interactions with the ubiquitin-like with PHD and RING finger domains protein 1 (UHRF1) [32]. More recently, Zhou et al. reported that a synthetic CG induces parthanatos via overexpression of poly-ADP ribose polymerase (PARP) and poly-ADP ribose (PAR) which are triggered by DNA damaging agents [33]. Our particular interest is in understanding their mode of action in the DDR signaling space as this is a common pathway dysregulated in cancer cells which holds considerable potential for therapeutic intervention [34–36].

The efficacy of many existing cancer therapeutics relies on the induction of DNA damage to kill cancer cells. DNA damage repair is activated to maintain genomic stability after DNA damage occurs. The DDR pathway is a signaling network that mediates DNA repair, damage tolerance processes, and cell cycle checkpoint pathways [37]. Hence, targeting and inhibiting the DDR pathway is a viable strategy in cancer therapy due to the reliance of cancer cells on DDR to mitigate the effects of replication stress. The anti-cancer activity of CGs has been demonstrated in some studies to target the DDR by regulating the expression and activity of certain proteins and kinases in the pathway to drive cancer cells into cell cycle arrest, apoptosis, or autophagy-dependent cell death [32, 38].

There is no record of a detailed summary featuring the mechanistic activity of CGs solely focusing on the DDR in the number of review publications outlining the progress of the anti-cancer mechanism of CGs [12, 39–46]. Therefore, this review will highlight the possible anti-cancer mechanisms affecting the DDR pathway that have been reported for different cardiac glycosides from 2010 to date in different cancers. We will focus on CG-induced DDR or DNA repair. Current advances and perspectives on the structural function in different CGs driving this activity will also be discussed. The scope of these molecules will be limited to glycosides with any backbone, including cardenolide or bufadienolide (Fig. 1b), as these have been the most well studied. Other steroidal compounds such as k-strophthadithin, antiarigenin, bufalin, and periplogenin will not be discussed as there are recent reviews covering this material [47, 48].

## Structural function and physicochemical properties of CGs

**Fig. 2:**
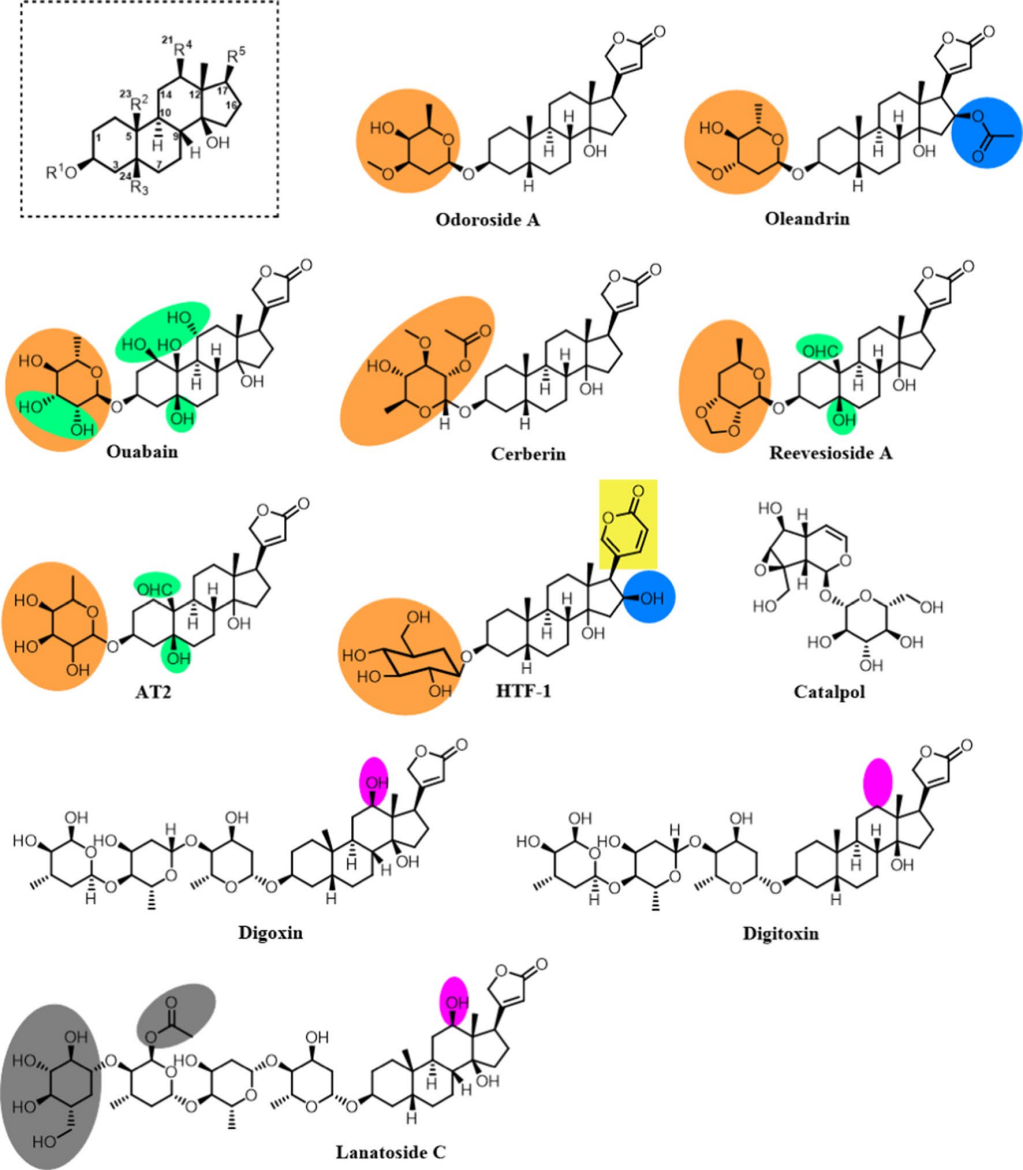
Summary of glycosides discussed in manuscript

The diversity of the structural size and chemical features of the various CGs illustrated in Fig. 2 begs the question: What are the important features that dictate different effects on cellular function? Cardiac activity is largely driven by interactions with the Na^+^/K^+^ ATPase, but the specific macromolecular targets for impacting cancer cell proliferation are still unknown.

Oleandrin is a derivative of odoroside A that possesses an acetate group at the C16 position of the aglycone that is absent in odoroside A (Fig. 2, blue highlight). As demonstrated by the activity of the two compounds, oleandrin displays a better cancer proliferation inhibitory activity than odoroside A [13]. The difference in activity of these two compounds can also be attributed to the difference in stereochemistry of the sugar moiety (Fig. 2, orange highlight). Additionally, the presence of the acetate group can increase the lipid solubility of the compound that consequently results in an enhanced chemical penetration through cellular membranes [13].

The structural motif of ouabain, cerberin, reevesioside A, and *Antiaris toxicaria* 2 (AT2) is similar to that of odoroside A. Ouabain has hydroxyl groups at the C4, C14, and C23 positions in place of protons as in the odoroside A. In addition to the differences in stereochemistry around the carbons in the sugar moiety, ouabain, like AT2, possesses additional hydroxyl groups around the sugar moiety (Fig. 2, green highlight). Conversely, cerberin and reevesioside A are sufficiently hydroxyl deficient. Cerberin has only one hydroxyl group in the monosaccharide with a couple of hydroxyl groups protected, whereas reevesioside A has no hydroxyl functional groups attached on the sugar moiety. However, reevesioside has a heterocyclic acetal bound to the sugar group. Furthermore, reevesioside A is modified at R^2^ with an aldehyde at position 23, differing from the other glycosides that have a methyl or hydroxyl group in that position. While each of these CGs have been demonstrated to possess anti-cancer activity, the SAR of these different glycosides has not been investigated in a single model. Thus, conclusive statements comparing their activity are difficult. However, we can predict a trend from a purely theoretical structural analysis of these CGs in the increasing order of lipid solubility, i.e., ouabain < AT2 < odoroside A ≤ cerberin < reevesioside A < oleandrin.

HTF-1 is a glycosidic molecule extracted and purified from *Helleborus thibetanus Franch* (HTF) plant [49]. HTF-1 is different from the other glycosides as it is the only bufadienolide. Bufadienolides have a pyrone ring at C17 whereas cardenolides have a furanone. Each of these compounds possesses some degree of anti-cancer activity suggesting that additional modification around C17 could be pursued to optimize this activity.

Catalpol is an iridoid glycoside found in *Rehmannia glutinosa.* It is comprised of a cyclopentane-pyran bound by a glycosidic bond to a glucose molecule. The activity of this compound, like other more known CGs, spans across multiple biological functions such as neurodegenerative diseases, diabetes, inflammation, and cancer [50]. Structurally, catalpol is drastically different from the conventional cardiac glycosides. Liu et al*.* [51] demonstrated that catalpol can influence the generation of reactive oxygen species (ROS) just as effectively as odoroside A or oleandrin. Structural analysis of these CGs postulates that the sugar moiety might be more responsible for ROS generation than the steroid core. This induction of ROS as well as DNA damage are common themes that will be discussed below as potential mechanism of CG-induced anti-cancer activity.

Digoxin, digitoxin, and lanatoside C possess *n-*sugar molecules. The significance of multiple sugar molecules was highlighted by Wang et al. [52]. In this study, the authors showed that cytotoxicity of cancer cells was better achieved by shortening the sugar chain. While multiple sugars would increase the overall solubility of the compound, it would lower its lipid solubility. This would affect the molecule’s membrane transport ability, consequently lowering its bioavailability in the cells/tumors. Compared to digitoxin, digoxin possesses a hydroxyl group at the C13 position instead of a proton (Fig. 2, pink highlight). Lanatoside C possesses four monosaccharides (Fig. 2). Like other glycosides with similar structural features, an examination of this position in digitoxin and digoxin would be worthwhile in understanding its function in CGs structures.

The above discussed glycosides have varying degrees of hydroxylation. Clearly, hydroxylation is an important motif in CGs that also needs to be addressed. As previously noted, lipid solubility has to be taken into account in order to ensure their absorption in cells and the body; therefore, some level of hydrophobicity must also be achieved. So, the questions we ask are: hat is an appropriate amount of hydroxylation? Also, is it relevant if hydroxylation is centered around the steroid frame instead of the sugar moiety? All the CGs except for ouabain (Fig. 2) show minimal hydroxylation on the steroid frame. But, if the vast interests and current applications of ouabain in research are any indication [24, 30, 53–55], hydroxylation of the steroid core might be an avenue worth exploring. However, one of the challenges with these bioactive compounds is the limited opportunity for functionalization, especially on the steroid core. Even in publications where a complete construction of the steroid core has been reported, a minimal degree of hydroxylation/oxygenation was achieved [19, 56, 57].

## Mechanism of CGs via DNA damage signaling

### Activation of DNA damage sensors

**Fig. 3:**
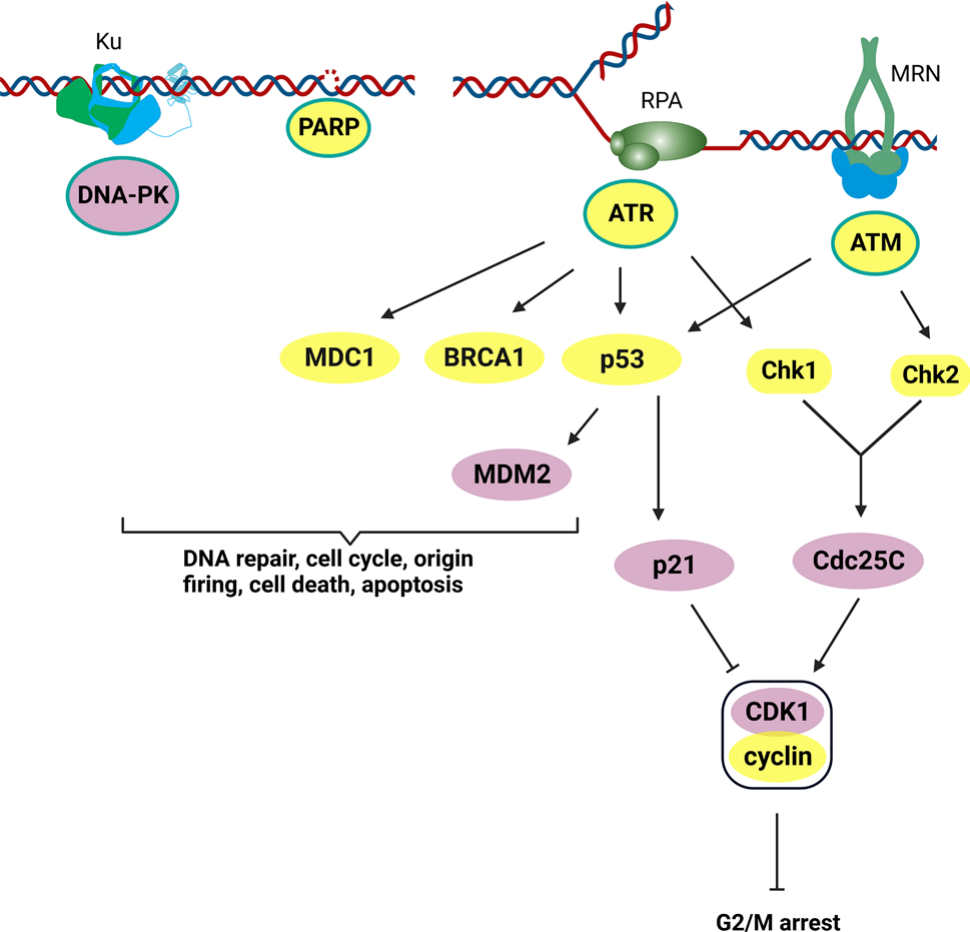
[1] DDR sensors and signaling. DNA damage including DSBs and SSBs, have been found following treatments with different CGs. (yellow = increased expression, pink = decreased expression) As such, upstream DNA repair proteins including PARP, ATM, ATR, and DNA-PK are recruited by DNA damage sensors RPA, MRN and Ku protein which triggers DDR signalling

DNA discontinuities are sensed by a series of proteins that then signal to regulate downstream effectors and pathways involved in DNA repair, cell cycle, and cell death. Ataxia-telangiectasia mutated (ATM) and DNA-dependent protein kinase (DNA-PK) are the two primary protein kinases that signal the presence of DNA double-strand breaks (DSBs). ATM is recruited by the MRE11-RAD50-NBS1 (MRN) DSB sensor, while DNA-dependent protein kinase catalytic subunits (DNA-PKcs) is recruited by the Ku70/80 heterodimer DSB sensor. Excess single-strand DNA associated with stalled replication forks is sensed by replication protein A (RPA) which signals to the ATM and Rad3-related (ATR) kinase, while single-strand breaks are sensed and signaled by PARP. CGs’ have been shown to impact each of these proteins. The CG ouabain has been studied as a potential anti-cancer agent in a variety of systems. A recent study demonstrated that ouabain can promote cytotoxicity in U2OS osteosarcoma cells via apoptotic cell death [55]. Another study validated this observation and implicated the DDR pathway [30]. Experiments performed using the comet assay and DNA electrophoresis revealed that ouabain induces DNA breaks and DNA fragmentation [30]. The authors also reported increased phosphorylation and expression of several DNA damage proteins such as ATM, ATR, p53, γ-H2A histone family member X (γ-H2AX), mediator of DNA damage checkpoint 1 (MDC-1), PARP, and breast cancer gene 1 (BRCA1) (Fig. 3). Additionally, Ouabain treatment suppressed the levels of DNA-PK and methylguanine methyltransferase (MGMT) gene expression as well as the phosphorylation of mouse double minute 2 (MDM2). The effect of ouabain on the signaling of ATM and ATR pathways is suggestive of CG-induced DSB and single-strand break (SSB) DNA damage. The limitation of this paper is the single time point (48 h after treatment) used in all the studies. Therefore, it is impossible to determine if the breaks measured were the direct effects of ouabain on DNA, an indirect effect on DNA and altered DDR, or simply the DNA fragmentation by the apoptotic nucleases and inconsequential changes in the DDR proteins of dying cells.

A more recent study of ouabain on different cancer cells performed time course studies and demonstrated increased ROS and DNA damage at time points before apoptosis [36]. A convincing study for a more direct effect was reported with CG (digitoxin) and HeLa cervical cancer cells, which reported induction of γ-H2AX at times before apoptotic chromosomal degradation [58]. They reported cell cycle alterations in the G_2_/M phase, and activation of the DDR pathway. This was notably marked by the phosphorylation of ATM and ATR. This study showed activation of the checkpoint kinases (Chk1 and Chk2) in addition to downregulation of cell division cycle 25C (Cdc25C). The evaluation of effector proteins, i.e., cyclin-dependent kinase 1 (CDK1) and cyclin B1 of the G_2_/M cell cycle phase was performed, and the results showed elevated levels of cyclin B1 while those of CDK1 declined. The investigation of digitoxin activity in this cell model provided evidence for activation of the mitochondrial apoptosis. This demonstration of early and potentially direct induction of DNA damage and activation of the DDR by a CG provides an explanation for the ability to combine with other DNA damaging therapeutics to enhance tumor cell killing.

The CG oleandrin has been shown to induce DNA damage in non-small cell lung cancer (NSCLC) A549 adenocarcinoma cells as measured by surrogate markers including RPA and γ-H2AX foci [31]. To examine what type of DNA damage repair is engaged in this oleandrin-induced apoptosis, the expression of Rad51 and X-ray repair cross-complementing protein 1 (XRCC1) was investigated. Rad51 and XRCC1 are markers of the repair of damaged DSBs and single-strand breaks (SSBs), respectively. Rad51 is a central catalyst in the homologous recombination (HR) pathway that protects stalled replication forks and mediates DSB repair. XRCC1 interacts with DNA ligase III and ligase I to facilitate SSB repair and base excision repair. The suppression of Rad51 was observed when cancer cells were treated with oleandrin, suggesting that the DSBs induced by oleandrin were likely not repaired by HR pathway, thereby resulting in cancer cell death. Conversely, the expression of XRCC1 was elevated which is indicative of activated XRCC1-induced SSB DNA repair.

### Activation of DNA repair sensors in combination studies with DNA damaging agents

In addition to single agent CG treatment demonstrating effective anti-cancer activity, combination studies have also been investigated in a few systems combining CGs with DNA damaging therapeutics including cisplatin, mitomycin C, camptothecin, and ionizing radiation (IR) [14, 21, 22, 32, 53, 59]. The targets again remain elusive, though combination treatments show impressive activity in a variety of cancer models.

Fanconi anemia (FA)/BRCA is a DNA repair pathway that is activated in response to DNA intrastrand crosslinks (ICLs). When the DNA repair process is activated in response to DNA damaging agents, the efficacy of the cancer treatment can be negatively affected, which in the long run could result in the development of drug resistance. This makes inhibition of the DNA repair pathway an attractive approach for enhancing the effectiveness of DNA damaging cancer therapeutics. In studies investigating the impact of CGs on FA/BRCA signaling in U2OS osteosarcoma cells, Jun et al. identified ouabain as a strong candidate for targeting this pathway [53]. The study demonstrated that this CG affected the protein expression of FANCD2 by transcription repression. The factors FANCF and excision repair cross-complementation group 4 (ERCC4) were repressed whereas the messenger ribonucleic acid (mRNA) levels of FANCA, Rad51, XRCC5, and XRCC6 remained constant. Comparable experiments performed with other CGs (digitoxin and digoxin) in this study showed similar activity to ouabain, demonstrating that the inhibition of FA/BRCA can be achieved with other CGs [53]. The mechanistic action of ouabain revealed the activation of p38 in its inhibitory function. The effect of ouabain was found to promote G2/M phase arrest only at high drug concentration. However, no effect was noted on the cycle progression at low concentration unless an ICL-inducing agent was present, at which point the S phase arrest was observed.

Lee et al. showed that digoxin not only enhanced DNA damage following IR treatments, but also reduced the levels of DNA repair proteins [60]. The study reported reduced expression and levels of Rad51, ERCC1, BRCA2, Ku70, Ku86, and DNA-PKcs in A549 cells but not H460 cells. These results suggested that digoxin inhibited the HR and non-homologous end joining (NHEJ) repair pathways in radioresistant A549 cells but not in radiosensitive (H460) cells. More recently, Wang and coworkers probed the anti-cancer activity of digoxin as a single agent and in combination with doxorubicin in NSCLC cell lines A549 and H1299 [59]. In their study, DNA damage and elevated ROS levels were detected after treatment with digoxin. The effect of this treatment on Rad51 and γ-H2AX expression resulted in an increase of γ-H2AX foci while inhibiting Rad51. Digoxin enhanced the expression of RPA and suppressed that of XRCC1. These results suggest that digoxin may inhibit the repair of SSBs and DSBs, though no direct measures of repair were reported. The complexity of the DDR and repair pathway necessitates measurements of repair proteins as expression levels are not an accurate measure of activity with many of the repair components being regulated by post-translational modifications. Tian et al. showed that AT2 was effective in combination with camptothecin, a DNA damaging agent that signals through Chk1 [32]. AT2 was shown to inhibit Chk1 activation and sensitize cells to a wide variety of clinically available chemotherapeutic drugs. The protein target of AT2 was identified as UHRF1 by quantitative mass spectrometry. UHRF1 is a multifunctional protein with roles in epigenetic maintenance and as a DNA damage sensor. Building upon that, we recently reported a series of chemical modifications of AT2 and their effects on impacting chemotherapy sensitivity in a lung cancer model [38], revealing innovative insights into the structure of CGs in inhibiting the DDR and DSB repair.

### Stress-activated signaling

The c-Jun N-terminal kinase (JNK) is one of the major DNA damage-activated protein kinases [61, 62]. This signaling pathway is triggered by both DNA damage and oxidative stress. JNK belongs to the mitogen-activated protein kinases (MAPK) family and promotes the phosphorylation of p53 that is also activated in the DDR process [63]. Activation of JNK triggers the upregulation of B-cell lymphoma-2 (Bcl-2) which inhibits the autophagy-inducing Beclin-1/PI3K-complex. Research involving CGs has demonstrated that JNK signaling can be stimulated as a result of treatment with CGs like HTF-1 and ouabain [49, 54]. Du and co-workers also reported the increased generation of ROS with CG treatment [36]. ROS are reactive oxygen species resulting from cellular oxidative metabolism and while they play a crucial role in several cellular signaling pathways, they can also provoke metabolic dysfunction. The mechanism by which ROS is generated is dependent on the specific sub-cellular compartment or organelle. ROS in the cytoplasm is formed using NADPH oxidase (NOX) proteins through nicotinamide adenine dinucleotide phosphate (NADPH) electron exchange [64]. In the mitochondria, ROS are created via oxygen interaction with flavin mononucleotide (FMN) [65] and reverse electron transfer (RET) [66]. ROS in the peroxisome is produced using oxidant scavenger enzymes which transfer electrons to water molecules to form hydrogen peroxide. An in-depth review article of ROS generation and their function in metabolic signaling is presented by Forrester and colleagues [67]. ROS can induce oxidative DNA damage and influence the DDR. Elevated levels of ROS can also increase the permeability of the outer membrane of mitochondria, inducing the release of pro-apoptosis factors.

**Fig. 4:**
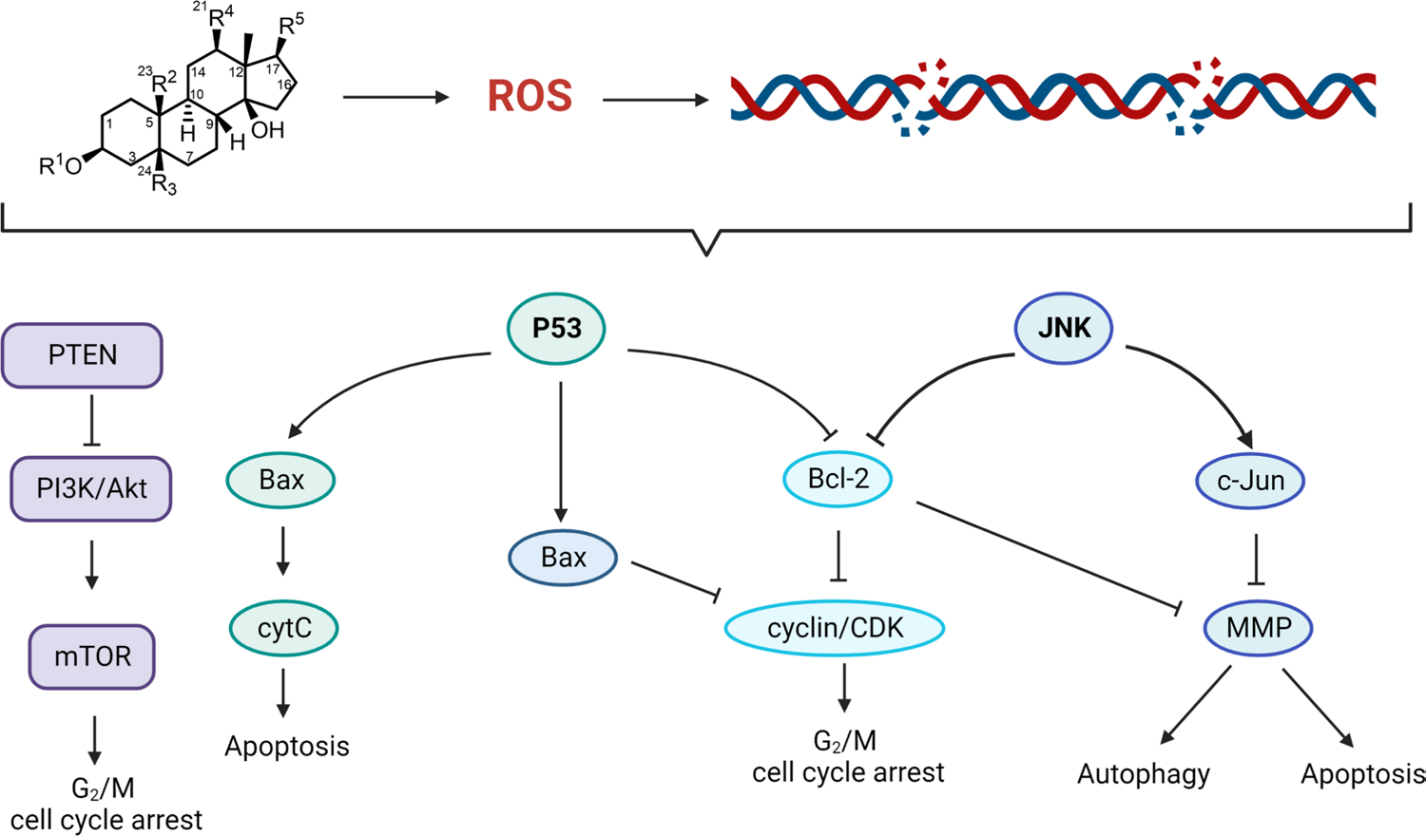
[1] CGs and ROS signaling. CGs trigger the production of ROS and DNA damage. A culmination of all or part of these consequences activate different signaling pathways; PI3K (purple), p53 (green) and JNK (blue)

The increased levels of ROS and the induced DNA damage as an effect of CG treatment resulted in activation of the JNK signaling pathway [36]. In their work, Hu and co-workers demonstrated that CGs (odoroside A and oleandrin) suppress the growth of acute myeloid leukemia (AML) cells by increasing the production of ROS which activates the phosphorylation of JNK [13]. This, consequently, triggered light chain 3B (LC3B) and caspase-9/caspase-3 that induce autophagy and apoptosis, respectively. The implication of caspase activation has also been investigated by other research groups [68]. Furthermore, these CGs inhibited the expression of Bcl-2 and matrix metalloproteinase (MMP) (Fig. 4). Bcl-2 is an anti-apoptotic gene and tumor promoter whose overexpression can inhibit the pro-apoptotic signal in cancer cells [69]. So, by deactivating Bcl-2, CGs can block Bcl-2-mediated DNA repair that is typically associated with genomic instability [70].

The role of CGs in the ROS formation and activity in apoptosis and autophagy has been extensively reported [13, 71–73]. Calderón-Montaño et al. reported that a *Nerium oleander* extract (NOE)-induced levels of ROS were significantly higher than the basal levels [21]. Additional experiments showed that ROS contributed to the cytotoxicity of NOE in lung cancer, colon cancer, and melanoma cells, albeit a minor role. Furthermore, cell treatment with NOE was shown to trigger DNA damage. A recent work demonstrated that ROS generation also mediated mitochondria apoptosis in breast cancer [51]. The study reported that catalpol not only inhibited the proliferation and growth of MCF-7 cells, but reduced the tumor volume in a xenograft model of breast cancer. The treatment with CG resulted in a decrease in MMP protein and an increase in ROS as well as levels of caspase-3 and cytochrome C (cytC). The upregulated levels of caspase-3 and cytC are indicative of mitochondrial apoptosis. Similar activity by oleandrin was also observed in colorectal cancer [74]. The results reported an upregulation of cytC and Bcl-2-associated X (Bax) protein that subsequently caused a downregulation of Bcl-2.

ROS-mediated DNA damage has also been shown to activate p53 signaling [75]. p53 is a tumor suppressor transcription factor that, when triggered, promotes the expression of kinases involved in apoptosis and cell cycle arrest. In their work, Chen et al. showed that CGs can promote G_2_/M cell cycle arrest and apoptosis via central events in the p53/ROS pathway. The increased population of cells in the G_2_/M phase is indicative of DNA damage [76]. Following treatment with odoroside A, an enhanced production of ROS was observed with suppressed expression of mutated p53. The reduced expression of mutated p53 not only caused apoptosis but also incited cell cycle arrest. Apoptosis was evidenced by an increase in expression of Bax, cleaved caspase-3, increase in cytC, and a noticeable decline of Bcl-2 (Fig. 4). The G_2_/M phase of the cell cycle arrest was shown to be activated by mutated p53 via regulation of p21 and cyclin/CDK complexes.

### PI3K/Akt/mTOR activated by CG-induced DNA damage

As one of the main growth regulatory pathways in both normal and cancerous cells [13], it is not surprising that the PI3K/Akt*/*(mammalian target of rapamycin)* mTOR* pathway (referenced as PI3K pathway) is influenced by the activity of CGs. Hyperactivated signaling of PI3K stimulates proliferation and reduces apoptosis in cancer, whereas inhibiting mTOR reduces cellular survival [77]. The cell survival pathways promoted by PI3K signaling is partly a function of impacting DDR [78]. This signaling pathway has also been implicated in DNA replication which suggests that inhibition of PI3K induces replication stress that consequently triggers DDR [79].

Studies showed that digoxin suppressed the cell cycle in the G_2_/M phase. While a study by Lindholm et al. [80] demonstrated that digitoxin had no effect on the signaling of PI3K/Akt pathway in numerous pancreas cancer cell lines, the majority of studies suggest a negative effect of CGs on the Akt/mTOR pathway. Treatment with digoxin inhibited the phosphorylation and expression of Akt and mTOR proteins in leukemia cell lines [81]. Although no evaluation was performed on the PI3K downstream factors, the observed apoptosis, cell cycle arrest, and subsequent suppressed progression of different cancers [77, 81] imply that mTOR downstream factors that cause apoptosis and cell death must have been activated by treatment with CGs. The downregulation of the PI3K pathway signaling was consistent with the work reported by others using cerberin, reevesioside A, and HTF-1 in different cancer cell lines [49, 82, 83]. The downregulation of components in this signaling pathway by CGs implicate them in triggering replication stress which leads to genomic instability and DNA damage.

In a different study, lanatoside C was shown to promote the upregulation of tumor suppressor genes (PTEN and p53). However, Akt, PI3K, mTOR, c-MYC, p62, LC3, beclin-1, and several cell cycle regulating protein kinases that include Chk1, Chk2, CDK6, and cyclin D1 were downregulated (Fig. 4) [84]. Furthermore, treatment with this CG also activated mitochondria-mediated apoptosis, G_2_/M cell cycle arrest and inhibited autophagy in breast, liver, and lung cancers. The results from this study also implicated lanatoside C in the activation of other signaling pathways such as JAK-STAT and Wnt/β-catenin that are involved in tumorigenesis.

### Activation of c-MYC/E2F

A non-apoptotic cancer cell death via disruption of the structure and function of the cell nucleus, downregulation of c-myc expression, and damage of cyclin-dependent kinases using a commercially available CG, UNBS1450, was an earlier plausible anti-tumor mechanism [85]. Cellular myelocytomatosis (c-MYC) is a transcription factor that plays a crucial role in cell growth, proliferation, and apoptosis. Overexpression of this gene induces oxidative DNA damage by increasing cellular metabolism and mitochondrial biogenesis [86]. Reevesioside A was found to cause mitochondrial damage and block cell proliferation in prostate cancer cell lines [83]. The activity of reevesioside A caused elevated E2F1 expression that is believed to be a result of DNA damage. This is suggestive of a reevesioside A-induced DNA damage. Even more relevant would have been an investigation of the DNA damage and the ROS content as exhibited by the action of this glycoside. The authors showed that reevesioside A downregulated the protein levels of c-myc as well as the expression of c-myc mRNA levels. This study further demonstrated that this CG could stall cell cycle progression at G1 phase by decreasing levels of cyclin D and CDC25A. The downregulation of c-myc using digoxin in triple negative breast cancer (TNBC) cells was also observed by Howard et al. [87]. This work further demonstrated that the inhibition of c-myc would result in reduced levels of eukaryotic initiation factor 4A-1 (eIF4A1).

## Perspective and conclusion

There has been a considerable increase in the investigation of the anti-cancer properties of cardiac glycosides, and several reports have provided insight into the anti-cancer mechanism of these compounds. As we have demonstrated above and summarized in Table 1, different signaling mechanisms are triggered when treatments with different CGs are made in different tumor cells. Without a doubt, we believe that the mechanism of anti-cancer action of CGs is likely a coalition of various signal transduction pathways. By analyzing and understanding these signaling pathways, we will be able to develop more targeted CG-mediated cancer therapies.

**Table 1:** Summary of the effect of different CGs on DNA damage and the DDR pathway

CGs	Effect on DNA damage and DDR	Refs
Odoroside A	Increased phosphorylation of JNK	[13]
Oleandrin and NOE	Induced ssDNA breaks and activate XRCC1-induced DNA repair pathway	[31]
	Promoted ROS formation	[21]
	Increased phosphorylation of JNK	[13]
	Promoted mitochondrial apoptosis	[74]
Ouabain	Increased expression and phosphorylation of HRR protein kinases such as ATM, ATR, p53, MDC-1	[30]
	Increased levels and expression of PARP and BRCA1	[30]
	Promoted DNA damage (ssDNA and dsDNA) and ROS formation	[30, 50, 54]
	Activated p38 signaling	[53]
	Suppressed the transcription factors FANCF and ERCC4	[53]
Cerberin	Downregulated levels of PI3K, Akt, and mTOR signaling	[82]
Reevesioside A	Caused mitochondrial damage	[86]
	Downregulated levels and phosphorylation of c-myc	[86]
	Downregulated levels of PI3K, Akt, and mTOR signaling	[83]
AT2	[in combination with camptothecin] targeted UHRF1	[32]
HTF-1	Downregulated levels of PI3K, Akt, and mTOR signaling	[49]
Catalpol	Promoted ROS formation	[51]
	Promoted mitochondrial apoptosis	[51]
Digoxin	Suppressed the transcription factors FANCF and ERCC4	[53]
	[in combination with IR treatments] Inhibited HRR and NHEJ repair proteins	[60]
	[in combination with doxorubicin] Elevated levels of ROS	[59]
	Suppressed the phosphorylation and expression of mTOR and Akt proteins	[77]
	Downregulated levels and phosphorylation of c-myc	[87]
Digitoxin	Increased expression and phosphorylation of ATM, ATR, chk1, and chk2	[58]
	Promoted mitochondrial apoptosis	[58]
	Suppressed the transcription factors FANCF and ERCC4	[53]
Lanatoside C	Promoted mitochondrial apoptosis	[84]
	Suppressed the phosphorylation and expression of PI3K, mTOR, Akt, MYC, and p62 protein kinases	[84]

Some of the most effective existing cancer therapeutics target DNA damage and the DDR pathway. Hence, it is not surprising that many studies of CG’s mechanistic role implicate DNA damage or repair. DNA damage and DDR signaling are promising targets for drug discovery because of their role in the progression and response in cancer therapy. Several studies have recognized the utility of CGs as potential agents that can increase the sensitivity of different cancer cells to other cancer therapies such as IR, doxorubicin, and cisplatin to mention a few [14, 32, 59, 77, 88]. This is an important stride in overcoming drug resistance and increasing the efficacy of these therapeutics. CGs have also been shown to cause various types of DNA damage such as DSBs, SSBs, and ICLs, whereas some forms of damage are exacerbated by the formation of ROS [13, 30, 31, 53]. Once induced, DNA damage activates signaling kinases that trigger responses including transcription, cell cycle arrest, and apoptosis. Additionally, CGs have been implicated in engaging the DNA repair pathways. For these reasons, it is important to closely investigate the activity of CGs in the DNA damage space.

Each of the four structural features (i.e., sugar moiety, glycosidic link, steroid frame, and lactone ring) of CGs have been identified to play a significant role in driving the efficacy of the compound from increasing the lipid solubility to serving a particular function, e.g., initiating apoptosis [52, 89]. However, using the evidence accrued from evaluation of the mechanistic activity of the different CGs discussed above, it is difficult to assign each structural motif to any particular function. What we can discern from this investigation are additional questions that must first be addressed to recommend new structural designs of CGs that are more potent. A degree of lipophilicity is necessary for the lipid solubility of these molecules; Does it matter if the hydroxylation is around the steroid core versus the sugar moiety? What is the role of the lactone? Is the unsaturation of that lactone ring relevant? How does the glycosidic link promote apoptosis? These questions can only be answered following a systematic SAR to identify the suitable features of CGs that enhance their activity. Our groups and several others have published different SAR results [20, 38, 52, 90]. However, a comprehensive investigation would be necessary to not only identify relevant structure moieties, but also discern which signaling pathways are activated and consequently trigger other pathways.

A key challenge in the successful development of glycosides as DDR inhibitors might arguably be their lack of specificity. The inherent complexity of the network of DNA damage signaling kinases and proteins is not the only obstacle. As discussed above, the glycoside-induced DNA damage and repair can trigger other signaling pathways such as growth factors, stress, and transcription [14, 24, 25, 84]. Consequently, we cannot exclude the possibility of an interplay of these signaling kinases as there are numerous studies of crosstalk (direct or indirect) between these pathways [91, 92]. To increase specificity, we must examine the role of these CGs in regulating signaling pathways. Only then can we begin to (1) identify the relevant targets to focus on and (2) design potent CGs for a specific function.

While we have provided a comprehensive analysis of the relevant data from which we can draw conclusions, we have to emphasize that there remain many unanswered questions. Definitive proof of the complete signaling pathways to explain the different mechanisms in the different cancers is lacking. We have explored and discussed the multiple DNA damage and DNA repair regulation proteins influenced by different CGs. However, we need more studies with various CGs to fully comprehend the mechanistic impact of CGs in activating these DDR-related proteins in order to allow the application and our understanding of these basic studies to direct and guide their clinical utility in cancer treatment.

## Data Availability

Not applicable.

## Abbreviations

Akt: Protein kinase B
AML: Acute myeloid leukemia
AT2: *Antiaris toxicaria* 2
ATM: Ataxia-telangiectasia mutated
ATR: ATM and Rad3-related
Bax: Bcl-2-associated X-protein
Bcl-2: B-cell lymphoma-2
BRCA1: Breast cancer gene 1
Cdc25C: Cell division cycle 25C
CDK1: Cyclin-dependent kinase 1
CG: Cardiac glycoside
CGs: Cardiac glycosides
Chk1: Checkpoint kinase 1
Chk2: Checkpoint kinase 2
c-MYC: Cellular myelocytomatosis
cytC: Cytochrome C
DDR: DNA damage response
DSBs: Double-strand break
DNA-PK: DNA-dependent protein kinase
E2F1: E2F transcription factor 1
eIF4A: Eukaryotic initiation factor 4A-1
ERCC4: Excision repair cross-complementation group 4
ERK1/2: Extracellular signal-regulated kinase ½
FA: Fanconi anemia
FANCA: Fanconi anemia group A
FANCD2: Fanconi anemia group D2
FANCF: Fanconi anemia group F
FMN: Flavin mononucleotide
FOXO1: Forkhead box O1
γ-H2AX: γ-H2A histone family member X
HIF-1α: Hypoxia-inducible factor 1 alpha
HR: Homologous recombination
HRR: Homologous recombination repair
JNK: C-Jun N-terminal kinase
ICLs: Intrastrand crosslinks
IR: Ionizing radiation
LC3B: Light chain 3B
MAPK: Mitogen-activated protein kinases
MDC-1: Mediator of DNA damage checkpoint 1
MDM2: Mouse double minute 2
MGMT: Methylguanine methyltransferase
MMP: Matrix metalloproteinase
MRN: MRE11-RAD50-NBS1
mRNA: Messenger ribonucleic acid
mTOR: Mammalian target of rapamycin
NADPH: Nicotinamide adenine dinucleotide phosphate
NHEJ: Non-homologous end joining
NOE: *Nerium oleander* Extract
NOX: NADPH oxidase
NSCLC: Non-small cell lung cancer
PAR: Poly-ADP ribose
PARP: Poly-ADP ribose polymerase
PI3K: Phosphatidylinositol 3-kinase
RET: Reverse electron transfer
ROS: Reactive oxygen species
RPA: Replication protein A
SAR: Structure–activity relationship
SSBs: Single-strand break
TNBC: Triple negative breast cancer
UHRF1: Ubiquitin like with PHD and RING finder domains 1
UNBS1450: 1R,3aS,3bR, 5aS,6aR,7aS,9R,12aR,13aR,15aR] -3a,11a-dihydroxy-13a-(hydroxymethyl)-9,15a-dimethyl-1-(5-oxo-2, 5-dihydrofuran-3-yl)icosahydro-1H,4′H-spiro[cyclopenta [7, 8]phenanthro[2,3-b]pyrano[3,2-e][1,4]dioxine-11,2′-[1, 3] thiazolidin]-4′-one
XRCC1: X-ray repair cross-complementing protein 1
XRCC5: X-ray repair cross-complementing protein 5
